# 

**Published:** 1809-10

**Authors:** 


### CORRESPONDENCE.

We have received two communications, containing very useful hints on CUPPING.—That of our Correspondent at Manchester is by some accident imperfect. If it is not too much trouble, we shall be thankful for a transcript.—The present copy shall be returned if it will be useful in refreshing the author's memory.

As Mr. Thompson expresses a wish to publish his paper with animadversions, we have returned it, and should his publication come before, he will not have occasion to accuse us of partiality.

